# Supplementary material for: Effects of Grape Polyphenols on the Life Span and Neuroinflammatory Alterations Related to Neurodegenerative Parkinson Disease-Like Disturbances in Mice
Source: Molecules. 2020 Nov 16;25(22):5339. doi: 10.3390/molecules25225339 (PMC7696792; doi:10.3390/molecules25225339)
Supplement: Supplementary file 1 [file molecules-25-05339-s001.pdf]

Supplementary Materials

Table S1. Content (mg/L) of active polyphenols in grape concentrate enoant (GPC) and dry wine material (DWM) from *Vitis vinifera* L. cv. "Cabernet Sauvignon" according to Zaitsev et al. (2010).

Substance	GPC	DWM
anthocyanins		
Delfinidin-3-O-glycoside	27.1	2.3
Cyanidin-3-O-glycoside	12.3	8.2
Peonidin-3-O-glycoside	13.0	12.6
Petunidin-3-O-glycoside	1.2	0.8
Malvidin-3-O-glycoside	167.5	135.7
Delfinidin-3-O-(6′-acetyl-glycoside)	12.4	9.4
Cyanidin-3-O-(6′-acetyl-glycoside)	3.6	2.2
Peonidin-3-O-(6′-acetyl-glycoside)	6.0	3.2
Petunidin-3-O-(6′-acetyl-glycoside)	2.3	1.2
Malvidin-3-O-(6′-acetyl-glycoside)	6.2	1.5
Delfinidin-3-O-(6′-n-coumaroyl-glycoside)	2.8	1.0
Petunidin-3-O-(6′-n-coumaroyl-glycoside)	6.5	1.6
Malvidin-3-O-(6′-n-coumaroyl-glycoside)	1.9	0.5
Flavones		
Quercetin	121.2	1.6
Quercetin-3-O-glycoside	46.0	3.9
Flavan-3-ols		
(+)-D-catechin	879.0	64.2
(-)-Epicatechin	567.0	32.1
(-)-Epicatechin-gallate	101.7	11.4
Hydroxycinnamic acids		
n-Coumaric acid	25.3	9.1
Caffeic acid	29.0	5.6
Trans-Coutaric acid	42.6	4.2
Trans-Caftaric acid	78.9	24.5
Hydroxybenzoic acids		
Gallic acid	928.4	135.7
Stilbenes		
Trans-Resveratrol	5.6	0.5
Oligomeric procyanidins		
Procyanidin B1	1857.0	267.0
Procyanidin B2	1325.0	155.0
Procyanidin B3	421.0	58.0
Procyanidin B5	269.0	45.0
Procyanidin B7	128.0	15.0
High-molecular weight and condensed procyanidins		
Total polymeric polyphenols	16370.0	2210.0

Zaitsev G.P., Catrich L.I., Ogai Yu.A. Biologically active polyphenols of the dry red 'Cabernet Sauvignon' wine material and the food concentrate 'Enoant'. Vinogradarstvo i Vinodelie (Magarach) 2010. 3: 25-27 (in Russian)

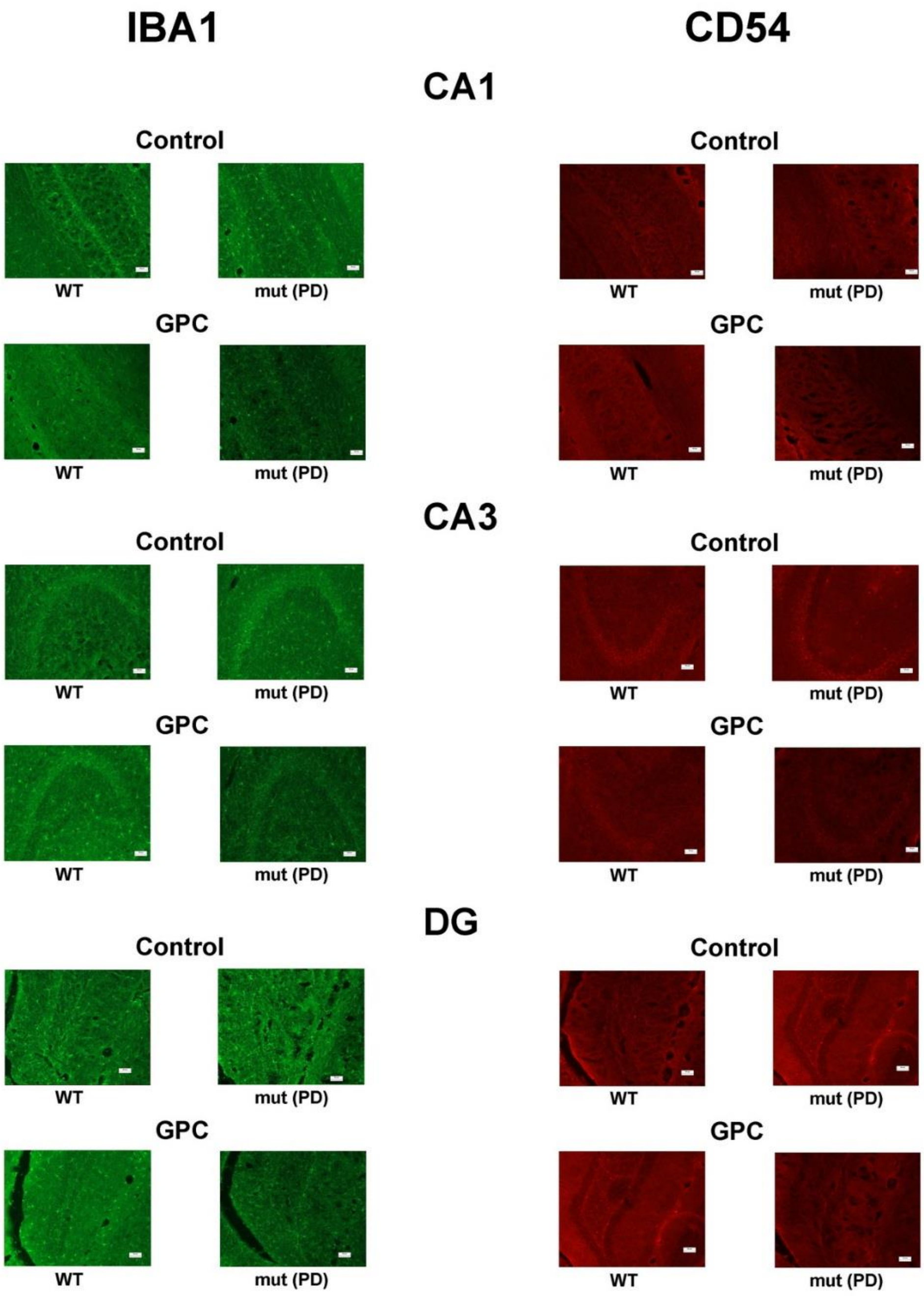

Figure S1. Effects of the overexpression of A53T-mutant α-synuclein and diet supplementation with *GPC* for four months on the immunoreactivity against the microglial marker IBA1 or inflammatory marker CD54 in the hippocampal CA1 area, CA3 area, and dentate gyrus (DG) in mice. Magnification, 200×; bar, 50 μm.
